# Practice scope and job confidence of two-year trained optometry technicians in Eritrea

**DOI:** 10.1186/s12909-019-1738-0

**Published:** 2019-08-07

**Authors:** Rajendra Gyawali, Bharat Kumar Bhayal

**Affiliations:** 1 Department of Optometry, Asmara College of Health Sciences, Post box no.: 1220, Mai Bela Ave, Asmara, Eritrea; 2 School of Optometry and Vision Science, University of New South Wales, Sydney, Australia

**Keywords:** Optometry technicians, Vision impairment, Eritrea, Primary eye care, Job confidence

## Abstract

**Background:**

A two-year optometry technician (OT) training was started in Eritrea in 2009 to fulfill the immediate human resource needs in providing refractive, dispensing and primary eye care services in vision centers. This study aimed to assess the current practice pattern and confidence level among the OTs.

**Methods:**

A self-administered questionnaire was developed and administered to all available OTs in January 2017. The OTs were identified through the Ministry of Health’s database. The questionnaire included questions on demographics, scope of practice and confidence level in the clinical practice areas.

**Results:**

A total of 94 OTs had graduated by the end of 2016 and 71 (75.5%) of them were involved in the country’s eye care services. All the 70 OTs who completed the survey were working under the Ministry of Health in various regions of the country. The mean age of the OTs was 25.6 ± 4.7 years (range: 20 to 48 years) and 43 (61.4%) of them were male. Four out of six regions in the country lacked the required number of OTs for the recommended ratio of one refractionist to 50,000 population. All the OTs provided refraction services; however, they lacked experience in dispensing (62.9%), clinical examination of patients (35.7%) and low vision care (4.3%). While the OTs expressed confidence in refractive procedures, low levels of confidence were expressed for dispensing and primary eye care services.

**Conclusion:**

OTs contributed to the primary eye care sector in Eritrea. However, high attrition rate, imbalanced distribution, a limited practice in core areas and low clinical confidence were the key challenges for this profession in this country. With better facilities, improved infrastructure and extended education and career opportunities, the two-year trained OTs could potentially serve further in the Eritrean eye care system. Further studies to evaluate the competency, job satisfaction and effectiveness OTs are recommended.

## Background

The most recent data on the global magnitude of vision impairment estimates that by 2020, there will be 237.1 million people with moderate to severe vision impairment and 38.5 million with blindness [1]. In Eritrea, the prevalence of blindness is estimated to be 1.5% with a further 10.5% suffering from moderate to severe vision impairment [2]. Similar to the global trend, cataract is the leading cause (56.3%) of overall vision impairment in the adults in Eritrea followed by uncorrected refractive error (18.1%) [2]. If these two major causes of vision impairment were considered priorities and control measures were implemented consistently across the world, two-thirds of the visually impaired people could recover good sight [1–4]. However, studies have shown a high rate of unmet need for refractive correction in both general adult population and visually impaired children in Eritrea [5, 6]. Lack of awareness, unavailability of services and high costs of spectacles have been cited as some of the reasons why Eritreans have very low met need of spectacles for refractive errors [5, 6]. Similarly, the lack of appropriate spectacles has also been shown to be responsible for poor visual outcome after cataract surgery in Eritrea [2]. Identifying the need for refractive services, a training course to produce mid-level ophthalmic professionals, named optometry technicians (OTs), was developed in 2009 [7].

The Diploma in Optometric Technician program was started in 2009 at Asmara College of Health Sciences with the support of Brien Holden Vision Institute and the Ministry of Health, Eritrea (MoH). These OTs are expected to provide category II services (Visual Function Services) based on the World Council of Optometry’s Global Competency-Based Model of Scope of Practice in Optometry [8]. The training and job scope of these OTs included refractive care, dispensing services, primary eye care including identification and referral for common ocular conditions, primary low vision rehabilitation services and community eye care [7]. Individuals who have completed high school education (grade 12) with physics, biology and mathematics as the core subjects can enroll in the program. Students are selected from a national pool of applicants based on their high school grades.

These students take two semesters of basic, biomedical and social science courses of total 33 credit hours (44.6% of total 74 credit hours) in the first year and then 40 credit hours (55.4% of total 74 credit hours) of professional courses in year two (Table 1) [8]. During the two-year course, students are exposed to didactic, practical and clinical activities to provide them with the necessary theoretical knowledge and practical skills to practice in hospitals and other eye care facilities. The clinical components are offered under the supervision of the College’s faculty, in affiliation with secondary level eye care centers and the national referral eye hospital in Asmara. After a satisfactory performance in these semesters (with a cumulative grade point average of > 2.0), students graduate the diploma course and join MoH for mandatory national service.

**Table 1:** Professional courses offered in third and fourth semesters (total credit hours = 74)^a^

Third semester Courses	Credit hours	Fourth semester courses	Credit hours
Ophthalmic Optics I	4	Ophthalmic Optics II	4
Physical and Geometrical Optics	4	Introduction to low vision	3
Dispensing Optics I	3	Dispensing Optics II	4
Ocular Anatomy and Physiology	2	Basic Ocular Disease	2
Physiological Optics	2	Public Health and Community Optometry	2
Clinical Optometry Procedure	4	Clinical Hospital Practice	3^b^

^a^One introduction to optometry course (3 credit hours) is also offered in second semester

^b^3 clinical credit hours is equivalent to 12 h per week of hospital placement

This survey was conducted to assess the current practice pattern, confidence level and perceived need for further training of OTs. The results from this survey are expected to assist in designing targeted continuing education programs in future and help inform a debate on the effectiveness of the training program.

## Methodology

A cross-sectional survey of all the OTs available in the country was conducted in January 2017. Ethical approval for the study was obtained from the Ethics Committee of Asmara College of Health Sciences. The college registrar’s database was used to obtain the details of enrolled students and the graduate OTs. Information on the OTs’ current official postings was obtained from the human resource department at MoH. Informed consent was obtained from all the participants before they proceeded to complete the printed questionnaire. The identified OTs were contacted in person or through a telephone call and invited to participate in the study. The majority of the participating OTs completed the survey before an informal cultural event at the college and for others, the printed questionnaire along with a cover letter was sent to their health facilities. The questionnaire was developed in self-administered format and in the English language.

The survey investigated the demographic details including the region of origin and current practice. There are 6 regions (called the *Zoba*s) which are the primary geographical and administrative divisions in the country. The other information sought in the survey included educational background; current work details including refractive services, job confidence and the perceived need for further training. The job confidence questionnaire was adapted from a previously validated questionnaire used in the evaluation of a similar workforce in India [9]. Based on their training areas and job description, several items in this questionnaire were added, such as vision center management, spectacle dispensing and vision screening skills. Overall, 22 job skills across 5 practice domains viz. refraction, clinical examination and diagnosis, primary eye care and management, dispensing services and community service and management were included. The participants were asked to rate their confidence level for each skill.

The data were managed and analyzed using the SPSS version 20 software. For job confidence, each skill confidence was measured in a scale of 0 (no confidence) to 3 (fully confident). For the purpose of analysis, responses 0 and 1 were considered ‘not confident’, while 2 and 3 were considered ‘confident’. We also calculated the mean confidence score of the participants for each skill. The confidence scores for each of the five domains were calculated as the percentage score of total domain score. As the maximum possible score in each skill item was 3, the total domain score was equal to the number of skills assessed in the domain multiplied by 3. Overall confidence score was calculated as the percentage score out of the total of all five domains. A multivariate logistic regression analysis was performed to examine the relationship between participants’ characteristics (age, sex, duration of the active job and practice *Zoba*) and their confidence in performing skills. In addition, Pearson’s correlation coefficient was used to assess the relationship between the domain confidence scores and age and duration of the active job. A *p*-value of less than 0.05 was considered statistically significant.

## Results

Since the beginning of the program in 2009, a total of 120 students enrolled and 94 (78.3%) completed the course by January 2017. Additional two students were expected to complete by the end of 2017. Out of these 94 graduates, 66 (70.2%) were male and 58 (61.7%) were from *Zoba* Maekel. At the time of the survey, 71 (75.5% of the graduate OTs) were involved in eye health services area in Eritrea; all except one were working under MoH. The following data represents the responses from the 70 government-employed OTs who agreed to participate and completed the survey.

The mean age of these OTs was 25.61 ± 4.7 years (range: 20–48 years) and 43 (61.4%) were male. More than two-thirds of them were from *Zoba* Maekel (48, 68.6%). The average duration of active optometry practice was 29.90 ± 22.2 months (range: 2–66 months). Table 2 presents the OTs’ current location (*Zoba*) of practice and a comparison with the World Health Organization recommended level of 1 refractionist per 50,000 population [10].

**Table 2:** Region (*Zoba*) wise distribution of OTs (*n* = 70)

Current practice *Zoba*	Number of OT (%)	Population (%)^a^	OT: population	Number of OTs required for the population^b^
Maekel	27 (38.5)	675,700 (19)	1:25025	14
Debub	13 (18.6)	952,100 (27)	1:73239	19
Gash Barka	9 (12.9)	708,800 (20)	1:78756	14
North Red Sea	7 (10.0)	653,300 (16)	1:93329	13
South Red Sea	5 (7.1)	83,500 (2)	1:16700	2
Anseba	9 (12.9)	549,000 (16)	1:61000	11
Total	70 (100.0)	3,622,400 (100)	1:51749	73

*OT* Optometry technician

^a^2005 estimate

^b^Based on WHO recommended ratio of 1 refractionist to 50,000 population

The practice pattern of the OTs is shown in Table 3. While 12 OTs (17.6%) were working at the national referral eye hospital in Asmara, others were involved in regional hospitals and vision centers across the country. There were 5 regional hospitals with cataract surgical services (led by an ophthalmologist or a non-ophthalmologist cataract surgeon) and 11 functional vision centers providing primary eyecare (led by an OT or ophthalmic officer). The OTs reported being routinely involved in refraction (100%), dispensing (62.9%); clinical examination of patients (ocular health examination) (35.7%); and diagnostic services including visual field examination (22.9%) among other areas. In their routine practice, 27 OTs (38.6%) also reported prescribing medications for ocular conditions such as conjunctivitis. The OTs reported working an average of 34.1 ± 8.8 h per week (range: 12 to 48 h) examining an average of 15 patients (range: 4–50) per day. Out of the total patients examined per day, one quarter (25.5%) were reported to be children and nearly half (47.14%) female. Out of all OTs, 63 (90%) performed both objective and subjective refraction before prescribing spectacle. Majority of them reported relying on auto-refractor (90%) and never performing cycloplegic refraction (57.1%) even when indicated. More than three quarters (76%) of the OTs reported encountering complex (high or irregular errors) refractive cases on a regular basis. Presbyopia was the commonest condition encountered (60.0% of the OTs) followed by myopia (15.7% of the OTs).

**Table 3:** Current practice pattern (*n* = 70)

Details of practice		Number of Optometry Technicians (%)
Type of health institute	National Referral Hospital	12 (17.1)
	Regional (*Zoba*) Hospitals	20 (28.6)
	Vision Centre	38 (54.3)
Involvement in specific practice area	Refraction	70 (100)
	Dispensing	44 (62.9)
	Clinical examination of patients	25 (35.7)
	Diagnostic examination	16 (22.9)
	Low vision	3 (4.3)
	Binocular vision/orthoptics evaluation/management	8 (11.4)
	Other areas (operation theatre assistance, minor surgery)	5 (7.10)
	Community outreach programs	8 (11.4)
	Prescribing medicines for eye diseases	27 (38.6)
Specific details in refraction practice	Performs objective refraction in all possible cases	64 (91.4)
	Uses only autorefractor for objective refraction	63 (90.0)
	Routinely prescribes glasses without subjective verification	7 (10.0)
	Performs cycloplegic refraction in indicated cases	15 (21.4)

The OTs expressed poor confidence in one or more skills in each job domains (Table 4). In refraction domain, the main lack of confidence was reported in refracting children and low vision skills. Over half of the OTs lacked confidence in any dispensing skills including dispensing of simple single-vision spectacles. They were also not confident in using instruments like slit lamps, ophthalmoscope, tonometer and detecting anterior or posterior segment disorders. Majority of them expressed a lack of confidence in managing ocular emergencies, although they were confident in providing a referral in appropriate cases. Similarly, over half of the OTs were also not confident in community screening and vision center management skills.

**Table 4:** Job confidence of the OTs (*n* = 70)

Job domain	Job skills	Number of confident OTs (%)	Confidence score (out of 3) ± SD	Mean domain percentage score ± SD
Refraction	Visual Acuity Assessment	69 (98.6)	2.9 ± 0.3	60.7 ± 12.0
	Retinoscopy	46 (65.7)	1.8 ± 0.9	
	Subjective Refraction	65 (92.9)	2.8 ± 0.6	
	Refraction in Children	15 (21.4)	1.0 ± 0.9	
	Low vision	12 (17.1)	0.7 ± 0.9	
Clinical Examination and Diagnosis	History Taking	57 (81.4)	2.5 ± 0.9	43.1 ± 19.3
	Slit lamp Examination	23 (32.9)	1.1 ± 1.0	
	Ophthalmoscopy	33 (47.1)	1.5 ± 0.9	
	Detection of Anterior Segment Anomaly	22 (31.4)	1.2 ± 0.9	
	Detection of Posterior Segment Anomaly	12 (17.1)	0.7 ± 0.8	
	Making a correct diagnosis	27 (38.6)	1.2 ± 0.9	
	Tonometry	19 (27.1)	1.0 ± 0.9	
Primary Eye Care and Management	Providing Primary management of common ocular conditions	27 (38.6)	1.3 ± 0.9	41.6 ± 17.9
	Providing appropriate referral	39 (55.7)	1.8 ± 1.0	
	Management of ocular emergency	10 (14.3)	0.7 ± 0.8	
Dispensing Services	Simple single vision spectacles	30 (42.9)	1.3 ± 1.1	39.8 ± 29.3
	Complex (high power) single vision spectacles	30 (42.9)	1.3 ± 1.1	
	Bifocal spectacles	27 (38.6)	1.2 ± 1.1	
	Progressive addition spectacles	20 (28.6)	0.9 ± 0.9	
Community Service and Management	Community Eye Health Education	36 (51.4)	1.5 ± 1.1	43.6 ± 31.6
	Screening services	30 (42.9)	1.3 ± 1.0	
	Vision Centre Management	22 (31.4)	1.1 ± 1.1	
Overall Practice				46.4 ± 14.2

*OT* Optometry technician, *SD* Standard deviation

Using binary logistic regression analysis, we examined the relationship between confidence on each job skill and participant characteristics including age, sex, current work *Zoba* and active job duration. No statistically significant relationships were observed except for the relationship between age of OTs and dispensing skills. The confidence of the OTs in different dispensing skills increased significantly with increasing age (single vision dispensing: odds ratio (OR) = 1.4, 95% confidence interval (CI):1.1, 1.9; complex single vision dispending: OR = 1.3, 95% CI:1.1, 1.7; bifocal dispensing: OR = 1.4, 95% CI:1.1, 1.9; and progressive spectacle dispensing: OR = 1.3, 95% CI:1.1, 1.7; *p* < 0.05 for all). There were no other statistically significant associations between the OT characteristics and the domain scores or the overall score. Although male OTs scored higher than females in all domains, the differences were not statistically significant (*p* > 0.05, t-test).

All the OTs also expressed a need for ‘refreshment training’ or a continued education session to revise their skill in most of the job domains. The main areas of interest for the refreshment training included disease diagnosis and management, slit lamp examination, refraction in children, dispensing of bifocals and progressive spectacles. Although none of the OTs reported considering changing their profession in future, all of them wished to be upgraded to a higher degree in optometry.

## Discussion

This study surveyed the optometric technician workforce and their confidence in performing clinical and related tasks using a self-administered questionnaire. The goal of the study was to assess the availability of OTs and their competency, in order to assist formulating a capacity-development response for strengthening national eye care system. The strength of the study is that it produces the first workforce analysis of any type of eye care professional in Eritrea using a universal sample. The results are expected to be useful for the authorities to identify the gaps in human resource management and eye care service provision in the country where avoidable vision loss is a significant public health challenge. However, there are some limitations to the study. The self-reported survey is likely to suffer from certain biases such as response bias where participants either over-state their skills to impress the interviewer or understate their skills to demand further training [11]. In addition, the survey instrument used was not validated in the Eritrean context. Further exploration of the issues identified in this survey such as high attrition, poor confidence, and job satisfaction in this important eye care cadre is recommended.

The findings show that although all of them are working in the area of refraction, less than two-thirds are involved in dispensing. The main reason for this was lack of functioning dispensing facilities at the practice areas. The MoH with assistance from Brien Holden Vision Institute planned to establish 39 satellite vision centers equipped with dispensing facilities. However, only 13 such vision centers were established across the country by the end of 2016 (unpublished document, planning meeting for the third national plan of action for control of blindness in Eritrea, December 2016). Even in the centers with dispensing facilities, lack of consumables, inefficient management and financial arrangements might have hindered OTs from practicing dispensing services. Similarly, only 3 OTs were involved in low vision services, and this involvement was in an ad hoc low vision clinic at the college. Although the training on low vision is basic, students learn skills to calculate the simple magnification required for near aids such as spectacle magnifiers [7]. A high unmet need for low vision rehabilitative services has been demonstrated in visually impaired children in Eritrea [5]. Currently, there is no formal low vision service available in Eritrea and the national blindness program needs to work on establishing various levels of low vision rehabilitative services where these trained OTs can be utilized.

Low involvement in disease diagnosis and management can be attributed to the fact that many of the OTs are working in secondary and tertiary centers where the existing ophthalmic officers are involved in patient examination and treatment of eye diseases. The other possible reason is that the training on ocular diseases is very basic and OTs are primarily trained to identify any abnormality and then refer to ophthalmic officers or ophthalmologists [7]. Some OTs working in rural parts without any ophthalmic officers, however, reported being involved in prescribing medications for the management of ocular infection such as conjunctivitis. It should be noted that the training on the management of the ocular disease is ambiguous and OTs are not trained to treat infection and other conditions except for certain emergencies such as suspected corneal ulcers. The effective use of any medications for the treatment of ocular disease including infections requires a thorough understanding of the disease, the pharmacodynamics and pharmacokinetics of the drug to be used and several other related factors [12]. In case of ocular infections, for example, the widespread use and inappropriate dosing of antibiotics without an accurate diagnosis, or the identification of therapeutic aims, may lead to toxicity, prolonged therapy, antibiotic resistance, and unintended harm to ocular structure [13, 14]. Deploying qualified eye care professionals including ophthalmic officers and optometrists to such remote locations, although challenging, could address this issue. However, these OTs can play a crucial role in the initial management of ocular emergencies such as ocular injury and corneal ulcer in rural communities [15]. Similarly, just over 10% of the OTs reported being involved in community outreach and screening programs. In countries like Eritrea where a large proportion of the population lives in remote areas, screening for conditions such as refractive error and cataract can help find cases and allow people to receive service at their own community [16]. Further, school vision screening programs help in timely identification of conditions such as uncorrected refractive errors and strabismus in children which need to be treated in the early stage to avoid amblyopia and vision impairment [17]. Such outreach programs are very limited in Eritrea and the blindness control program should consider such programs utilizing already existing workforce including the OTs.

The second national strategic plan of action for the control of blindness in Eritrea set a target to produce 50 OTs by 2015. This target was met in the year 2016 with the production of 94 OTs of which 71 were active in the Eritrean eye care system. These numbers suggest that Eritrea has almost achieved the number of refractionists recommended by WHO (1 refractionist for 50,000 population) [10]. Although it was not possible to obtain official details of those who are not working in the eye care sector, informal communication with their associates shows that they have either fled the country or embraced other professions. In many developing countries, especially the low-income African nations, high attrition of skilled health care professional is a significant challenge in eye care systems, and this is associated with underdeveloped health systems in the home country and attraction from developed countries with better economic opportunities [18–22]. Factors such as better remuneration, ideal working conditions abroad, high-stress levels, increased workload and reduced job satisfaction have been long blamed for an exodus of healthcare workers from developing countries to places where their expectations are met [23]. Specific to mid-level ophthalmic professionals, inappropriate job skills match and no career structure have been reported as factors for demotivation and attrition, for which strategies such as standardized training and career advancement opportunities have been recommended [24]. Similarly, confusion about job responsibilities, lack of planning and policies, lack of infrastructure and funding to supplement production have also been identified as contributing factors [25]. In the Eritrean context, although there might be a political reason for such large migration [26], job satisfaction could also be a significant factor. In poor countries, poor working conditions and low remuneration are often the cause of such low job satisfaction. For example, the basic salary of an OT in 2016 ranged from 600 to 750 Eritrean Nakfa (equivalent to 40 to 50 US Dollars) per month. Recent government efforts to raise the salary scales based on the academic qualification and years of work is a positive development. As we were unable to interview those who had fled the country or embraced other professions, exact reasons for such high attrition cannot be confirmed. While waiting for further studies to identify reasons for this high attrition, the MoH should take actions such as increasing facilities, providing career development opportunities and improved workplace conditions to retain this valuable human resource.

In addition to the cross-border migration, inequitable distribution of health care professional is one of the greatest challenges in the health care system of a developing country [27]. Many eye care professionals in both developed and developing countries are concentrated in urban areas creating an imbalance in service demand and provision [28, 29]. Although many countries have increased their health workforce significantly, this has not addressed the health care need of their rural communities [30–32]. This gross inequitable geographical distribution of eye health workforce is also reflected in our study. While it is expected that the central region of *Zoba* Maekel (where the capital city Asmara is located) has a higher share of the OTs due to a larger population and the presence of the national referral eye hospital, other *Zoba*s had a disproportionate number of the OTs. The national referral eye hospital alone had over 17% of the OTs and an additional 21% within other vision centers in capital Asmara. Although the number of OTs in Maekel and South Red Sea regions appear sufficient, 21 additional OTs will be required to provide service in the other *Zobas* to meet the World Health Organization recommendation [10].

In addition to increasing the number of OTs to mitigate this workforce shortage, other strategies are also required to meaningfully address the eye care need of rural communities. As most of the *Zobas* have limited number of health care facilities (secondary hospitals with eye departments and vision centers) and these are primarily located in urban or semi-urban location, placement of the additional OTs in such facilities is only going help little, or at worst be a challenge for human resource management for a small facility. For example, a vision center located within a community health center in northwest Asmara had 7 OTs. However, because of limited working hours (outpatient department in Eritrea usually run in morning hours only) and facilities (for example examination room, chairs and instruments), only 3 to 4 OTs worked each day, with others taking the day off. Implementing the MoH plan to establish and functionalize 39 vision centers across the country, including those in rural areas, could help in proper placement of these OTs and improve the accessibility to refractive services for the rural and remote communities. The other key strategy can be addressing the disparity in access to education [30], as it has been suggested that optometry graduates from a rural background are more likely to work in rural setup after completing their education than those from urban background [33]. The economic reasons for more urban orientation, previously shown amongst optometrists [32, 34] and other health care professionals [27], also need to be addressed. This may be achieved through additional facilities, incentives, career opportunities and continued education for those in rural areas.

In terms of clinical confidence, OTs expressed low but varied confidence levels across all domains with the lowest confidence in dispensing. They reported low confidence in all the dispensing skills including simple single vision spectacles dispensing. The low confidence in dispensing is particularly concerning not only because of the role they are expected to play in providing dispensing services but also because it is a core skill taught in the program. This low confidence was likely due to the lack of dispensing facilities in many of the health care facilities where these OTs worked. On a positive development, several new vision centers had started providing basic dispensing services towards the end of 2016. This could help improve the practice pattern and confidence level among the OTs. Further, as the OTs also expressed, there needs to be a refreshment training in dispensing to ‘brush-up’ their skills and the vision centers with dispensing facilities need to start functioning to ensure people’s access to the affordable spectacles.

The confidence score in refraction was the highest among all the domains. However, the OTs reported poor confidence in refracting children. The training time and scope for the OTs in pediatric refraction are limited and as the refractive procedure and prescription in children can be daunting and complex than the adults, this lack of confidence is understandable. In informal discussions with the OTs working in regional hospitals and vision centers, they expressed the concern about children with binocular vision disorder and refractive error who need to be referred to Asmara for examination with pediatric optometrist and ophthalmologist. This usually results in a huge financial burden to the family and in many cases, the families cannot afford such a journey and the child’s vision impairment remains uncorrected. With the completion of the first batch of five-year trained degree optometrists in 2018 and their placement in these health facilities, such challenges can be expected to be addressed to some extent. However, it may be wise to provide the OTs from rural parts basic training in pediatric refraction and binocular vision to address the immediate need. Many of the OTs also indicated this area as their preferred topic for continued training programs. Pediatric refraction should be included in the future versions of the curriculum to provide more competency focused training.

Similarly, poor level of confidence was observed in the primary management of common ocular conditions; especially the management of ocular emergencies. As many of these OTs are working with ophthalmic officers who handle the ophthalmic outpatient and emergency department of the health facility, the OTs reported not being involved in evaluation and management of the ocular emergency such as trauma, corneal ulcers and acute painful red eyes. With future expansion and establishment of the vision centers, where these OTs might need to work alone, they need to refresh their skills in first aids of some specific emergency such as corneal ulcers. This also applies for the clinical examination skills such as ophthalmoscopy, slit-lamp biomicroscope and detection of prevalent anterior and posterior segment anomalies (for example trachoma, glaucoma and diabetic retinopathy).

The concept of vision centers in Eritrea envisages an OT as the in-charge of the center and thus management skills such as managing available resources, other staffs and the finances are thought to be important. Although there are only a few vision centers currently being led by OTs, it was interesting to observe that nearly half of the OTs expressed confidence in their abilities to manage a vision center. On the other hand, nearly 60% of the OTs were not confident in providing screening and outreach services in their communities. Lack of exposure to such activities during training and limited outreach services conducted in the country may have been responsible for this low confidence. As mentioned earlier, implementation of outreach programs will not only help in the detection and management of eye conditions but also improve the confidence of the OTs in such activities.

## Conclusion

Two-year trained optometry technicians are contributing to the eye care sector in Eritrea. However, there is a high rate of attrition rate from the profession and imbalance in their distribution within the country. They have limited practice in the core training areas especially dispensing and primary eye care. Their confidence in specific skills like pediatric refraction, dispensing spectacles, and primary management of ocular emergencies is low. Refreshment training in the core practice areas with poor confidence is a felt and observed need. With better facilities, improved infrastructure and extended education and career opportunity, the two-year trained OTs can potentially contribute further in the eye care system of Eritrea. Further studies to evaluate their clinical competency, job satisfaction and effectiveness in Eritrean eye care system are recommended.

## Data Availability

The datasets used and/or analyzed during the current study are available from the corresponding author on reasonable request.

## Abbreviations

MoH: Ministry of Health, Eritrea
OT: Optometry Technician
